# Investigation of bacterial effects of Asian dust events through comparison with seasonal variability in outdoor airborne bacterial community

**DOI:** 10.1038/srep35706

**Published:** 2016-10-20

**Authors:** Jonguk Park, Tomoaki Ichijo, Masao Nasu, Nobuyasu Yamaguchi

**Affiliations:** 1 Graduate School of Pharmaceutical Sciences, Osaka University, 1-6 Yamada-oka, Suita, Osaka 565-0871, Japan; 2 Faculty of Pharmacy, Osaka Ohtani University, 3-11-1 Nishikiori-kita, Tondabayashi, Osaka 584-8540, Japan; 3 Osaka Prefectural Institute of Public Health, 1-3-69 Nakamichi, Higashinari, Osaka 537-0025, Japan

## Abstract

Atmospheric bacterial dispersion with aeolian dust has been reported to have a potential impact on public health and ecosystems. Asian dust is a major aeolian event that results in an estimated 4 million tons of Asian dust particles falling in Japan annually, 3,000–5,000 km away from their source regions. However, most studies have only investigated the effects of Asian dust during dust seasons. Therefore, in this study, outdoor bacterial abundance and community composition were determined by 16S rRNA quantitative PCR and amplicon sequencing, respectively, and compared on Asian and non-Asian dust days (2013–2015; 44 samples over four seasons). Seasonal variations in bacterial abundance of non-Asian dust days were not observed. Bacterial abundance of individual samples collected on non-Asian dust days changed dynamically relative to Asian dust days, with bacterial abundance occasionally reaching those of Asian dust days. The bacterial community composition on non-Asian dust days was rather stable seasonally, and did not differ from that on Asian dust days. These results indicate that bacteria in Asian dust does not immediately influence indigenous bacterial communities at the phylum/class level in distant downwind areas; accordingly, further studies of bacterial communities in downwind areas closer to the dust source are warranted.

Aeolian dust is a natural phenomenon that primarily occurs in arid and semi-arid regions such as deserts and areas with loess. In this phenomenon, sand particles are lifted by ascending air currents and transported over long distances (hundreds or thousands of kilometers). Approximately 0.5–5.0 billion tons of aeolian dust are transported each year worldwide^1^. Major aeolian dust events arise from the Sahara and Sahel deserts (African dust), the Australian deserts (Australian dust), and the Taklamakan and Gobi deserts and the Loess Plateau (Asian dust).

Atmospheric currents and ocean currents are the main vehicles of bacterial migration, and it has been reported that bacteria adhered to aeolian dust particles may impact public health and ecosystems^2^,^3^,^4^,^5^. It is estimated that approximately four million tons of Asian dust particles fall on Japan each year^6^, which is 3,000–5,000 km away from their source regions. In addition, Asian dust particles sometimes reach North America, more than 15,000 km from their source regions^7^,^8^. Several investigations of airborne bacteria have been conducted to evaluate potential health effects of long-distance transport of bacteria by Asian dust, including analyses of bacterial community composition^9^, abundance and viability^10^, as well as investigations of atmospheric halotolerant bacterial communities^11^.

Previous studies of aeolian dust using culture-dependent methods have reported the airborne transport of pathogens and their resultant health effects^4^,^8^. However, community analyses using culture-independent methods have confirmed that aeolian dust transported not only pathogenetic bacteria, but also phylogenetically diverse bacteria^9^,^11^,^12^,^13^,^14^. In addition, most studies have been conducted only during Asian dust events (March to June)^9^,^10^,^11^,^12^,^14^, even though the bacterial abundance and community composition of aerosols in outdoor environments are thought to be affected by seasonal and weather-related variations^15^,^16^,^17^,^18^,^19^. To assess the effects of bacteria transported with aeolian dust on public health and the environment, bacterial variations should be evaluated by long-term monitoring.

Therefore, the present study was conducted to investigate the effects of bacteria transported by Asian dust events on humans and the ecosystem based on outdoor aerosol samples collected on both Asian dust and non-Asian dust days from 2013 to 2015. We analyzed variations in bacterial abundance and bacterial community composition on non-Asian dust days to understand variations in the local airborne bacterial community. We then investigated airborne bacterial community characteristics following Asian dust events through comparison with seasonal bacterial community variability on non-Asian dust days and changes in the bacterial community on Asian dust days. Airborne bacterial abundance and community composition were determined by 16S rRNA gene-targeted quantitative PCR and amplicon sequencing, respectively.

## Results

### Variations in bacterial abundance on non-Asian dust days and comparison with bacterial abundance on Asian dust days

**Figure 1:**
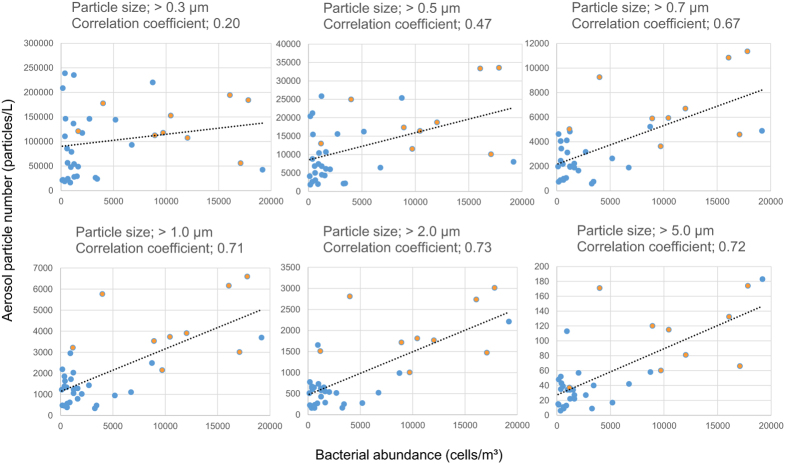
Correlation of bacterial abundance with particle size distribution. The bacterial abundance was determined by quantitative PCR targeting the 16S rRNA gene (V6–V8). Blue and yellow indicate non-Asian and Asian dust samples, respectively.

Particle size distribution of aerosols in the outdoor environments was measured (Supplementary Fig. S1). The number of particles in the outdoor environment was changed by 10 fold. During Asian dust events, particle number was generally elevated, and comparatively large particles were dynamically elevated (P value; > 0.3 μm [0.0255], >0.5 μm [0.0069], >0.7 μm [0.0011], >1.0 μm [0.0003], >2.0 μm [0.0002], >5.0 μm [0.0028]). The results revealed a correlation between bacterial abundance and particle size distribution, with particle sizes larger than 1.0 μm showing a greater correlation (Fig. 1).

**Figure 2:**
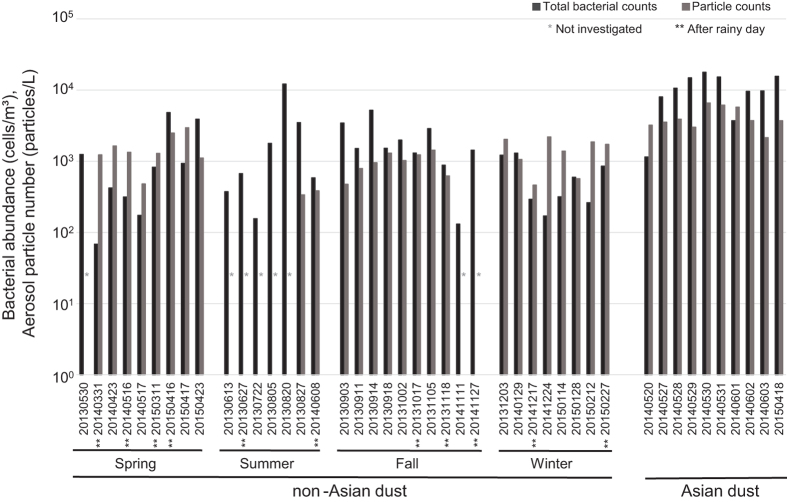
Total bacterial abundance (black bar) determined by quantitative PCR targeting the 16S rRNA gene and total aerosol particle numbers (particle size: > 1.0 μm [gray bar]) determined by particle counter. Samples were collected during each of the four seasons, including after rainy days and Asian dust days.

Accordingly, we compared bacterial abundance and number of particles larger than 1.0 μm during different seasons, rainfall events, and Asian dust occurrences (Fig. 2). The levels of particles larger than 1.0 μm fluctuated between 3 × 10^2^ and 3 × 10^3^ L^−1^, regardless of season. On Asian dust days, particle levels ranged from 2 × 10^3^ to 7 × 10^3 ^L^−1^. Particle numbers on Asian dust days were higher than those on non-Asian dust days, and their fluctuation was more stable on Asian dust days.

Bacterial abundance in outdoor environments varied with variations in particle number (Fig. 2; 1 × 10^2^–1 × 10^4^ cells m^−3^), and bacterial abundance was not influenced by rainfall in this study. However, bacterial abundance generally increased as the number of particles (>1.0 μm) increased, without response to seasonal variations or occurrence of Asian dust (correlation coefficient; r > 0.7). Bacterial abundance on Asian dust days was generally greater than 10^4^ cells m^−3^. The average bacterial abundance on Asian dust days ([1 ± 0.6] × 10^4^ cells m^−3^) increased by approximately 5 times relative to non-Asian dust days ([2 ± 3] × 10^3^ cells m^−3^). However, bacterial abundance fluctuated from 10^2^ to 10^4^ cells m^−3^ on non-Asian dust days and changed dynamically relative to Asian dust days, with bacterial abundance reaching that of Asian dust days on several occasions (20 August, 27 August, 3 September, and 14 September, 2013; 16 April and 23 April, 2015).

The ratio of bacterial abundance to number of particles (>1.0 μm) was comparatively higher in summer and fall (0.56% and 0.28%, respectively). However, this ratio was lower in winter than in other seasons (0.15% in spring, 0.06% in winter). On Asian dust days, the ratio of bacterial abundance to particle number was 0.30% and stable relative to non-Asian dust days.

### Variations in bacterial community composition on non-Asian dust days and comparison with bacterial community composition on Asian dust days

To investigate the bacterial effects of Asian dust, we also analyzed bacterial community composition with variations in environmental conditions on non-Asian dust days. Airborne bacterial community composition in outdoor environments has been reported to change in response to variations in environment factors^15^,^16^,^17^,^18^,^19^. In this study, the airborne bacterial community composition was determined using 16S rRNA gene targeted ion PGM sequencing in conjunction with a two-step PCR method. Two-step PCR has advantages such as increased reproducibility and recovery of higher genetic diversity during amplicon sequencing^20^,^21^.

**Figure 3:**
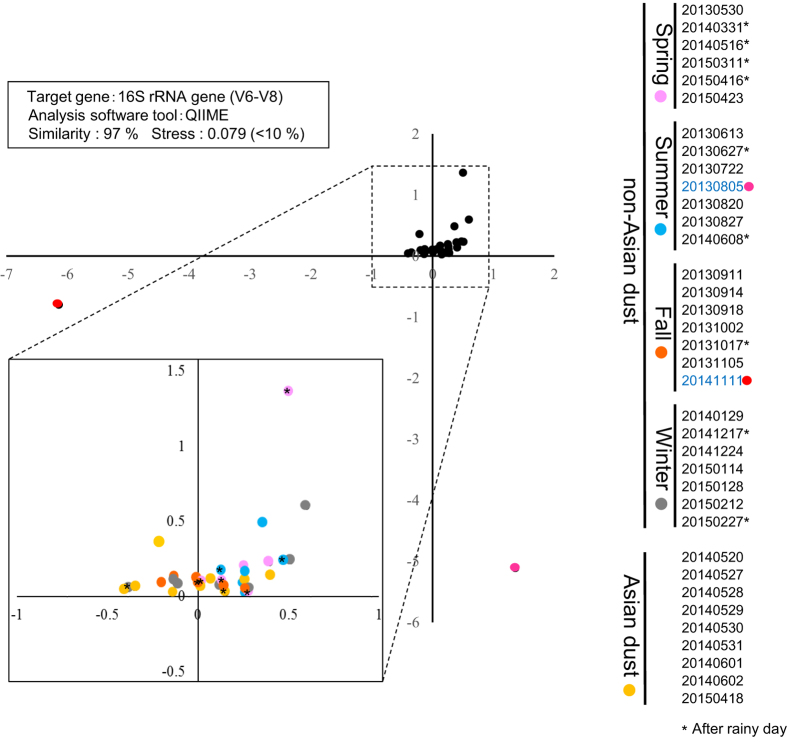
Multidimensional scaling (MDS) analysis of bacterial 16S rRNA genes obtained from aerosols in outdoor environments.

**Figure 4:**
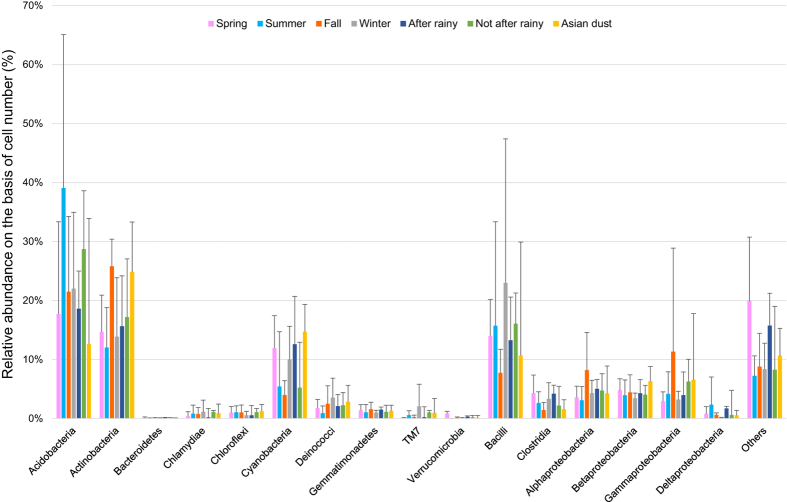
Relative abundance of each common phyla and class in outdoor samples (classified by season, weather conditions, and Asian dust events).

In the two-step PCR method, we used the 968f–1401r primer (V6-V8) set because it produced the highest diversity in a preliminary study using PCR-DGGE to select the proper primer (Supplementary Fig. S2). To analyze similarities in the bacterial community composition of each sample, amplicon sequencing data of bioaerosol samples were processed using the QIIME software, and the results were indicated using multidimensional scaling (MDS) (Fig. 3). The results revealed that bacterial community composition in the outdoor environment was rather stable, despite changes in season, and samples were generally not affected by variations in environmental factors. However, the bacterial community compositions of samples collected on 5 August 2013 and 11 November 2014 differed from others. In addition, bacterial community composition did not differ significantly on Asian dust days and non-Asian dust days. Comparative analysis of the bacterial community composition at the phylum and class level revealed that environmental factors such as season and rainfall had no effect on the predominant bacterial phylum and class (Fig. 4).

**Figure 5:**
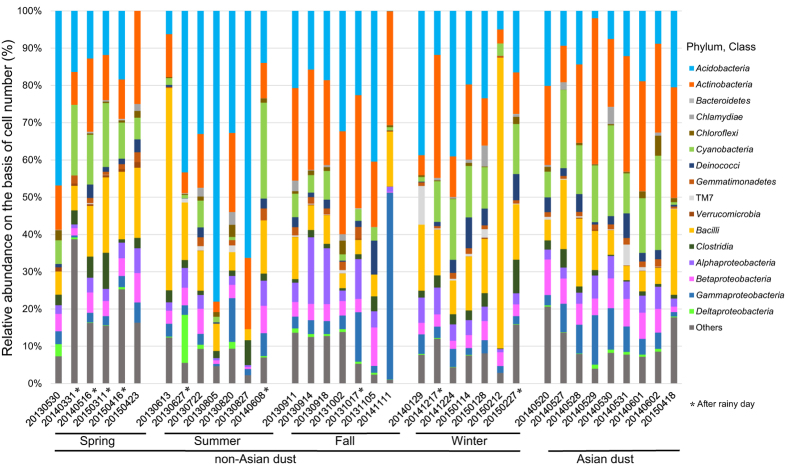
Taxonomic composition of each sample of outdoor airborne bacterial communities. Composition estimates are based on relative abundances of bacterial 16S rRNA gene sequences assigned to different common phyla and classes.

The predominant phyla and classes on non-Asian dust days were *Acidobacteria* (25 ± 19%), *Actinobacteria* (17 ± 9%), *Bacilli* (15 ± 16%), *Cyanobacteria* (8 ± 7%), *Alphaproteobacteria* (5 ± 4%), *Gammaproteobacteria* (5 ± 9%), *Betaproteobacteria* (4 ± 2%), *Clostridia* (3 ± 2%), and *Deinococci* (2 ± 3%) (Fig. 5). No specific phylum or class accounted for more than half of the total bacterial community. Variations in the bacterial community composition between seasons did not exceed more than double the original concentration. On Asian dust days, the bacterial community composition was similar to that on non-Asian dust days, with dominant members including *Actinobacteria* (25 ± 9%), *Cyanobacteria* (15 ± 8%), *Acidobacteria* (13 ± 6%), *Bacilli* (11 ± 7%), *Gammaproteobacteria* (7 ± 4%), *Betaproteobacteria* (6 ± 2%), *Alphaproteobacteria* (4 ± 2%), *Deinococci* (3 ± 2%), and *Clostridia* (2 ± 1%) (Fig. 5). Changes in the outdoor airborne bacterial community composition in response to Asian dust did not exceed those observed on non-Asian dust days.

On 5 August, 2013, the bacterial community composition differed from that observed on other sampling dates, with *Acidobacteria* being the dominant member (78%). *Acidobacteria* are generally the dominant phyla in soil habitats^22^. Additionally, on 11 November, 2014, *Gammaproteobacteria*, which is known to exist in diverse environments, was dominant (50%). *Bacilli* can form spores and withstand severe conditions such as those found in sources of Asian dust^23^. There have been many reports of increased levels of *Bacilli* on Asian dust days in downwind regions far from the source regions of Asian dust^9^; however, *Bacilli* accounted for more than 50% of the population on several non-Asian dust days in this study (13 June, 2013 [54%], 12 Febraury, 2015 [78%]). *Bacilli* did not increase in response to Asian dust events in this downwind area.

## Discussion

In this study, bacterial number and community composition were calibrated based on copy number of 16S rRNA gene of each bacterial phylum. This was conducted because both high copy number bacteria (e.g., *Bacilli*) and low copy number bacteria (e.g., *Acidobacteria*) were present in the collected dust samples.

Although bacterial abundance has been reported to change in response to variations in environmental factors^15^,^16^,^17^,^18^,^19^, it was not correlated with any environmental factors (season, temperature, humidity, wind speed, wind direction, rainfall) except particle numbers in the present study. During winter, the ratio of bacterial abundance to particle number was low relative to other seasons. Atmospheric bacterial abundance would be lower in winter because of the response to low temperature^24^.

No considerable increase in bacterial abundance was observed on Asian dust days relative to fluctuations in bacterial abundance on non-Asian dust days. Bacterial abundance on aerosols of indoor environments usually ranges from 10^5^ to 10^6^ cells m^−3 ^25, and bacterial abundance in outdoor environments determined in this study was 10–100 times lower than those in indoor environments.

The results on non-Asian dust days appeared to be correlated with the environmental characteristics of the sampling location. Specifically, we monitored bioaerosols in environments in which temperatures are suitable to the growth of general bacteria (from 4 °C to 34 °C; average 21 ± 9 °C). It has been reported that bacterial abundance and community composition changed significantly in response to season in specific places (e.g., coastal sites^15^, high-elevation sites^16^). However, none of our sampling points were located in places such as these. Variations in atmospheric bacterial community composition in outdoor environments impacted by Asian dust occurrence were more stable than those observed on non-Asian dust days. Accordingly, these findings indicate that bacterial effects on humans and ecosystems in distant downwind areas impacted by Asian dust may be lower than those of general changes in the natural environment.

Information describing the viability of airborne bacteria collected on Asian dust days can help accurately estimate their influences on public health and ecosystems. Several methods, such as fluorescent vital stain^10^, can be used to estimate bacterial activity in aquatic and soil environments. However, bacterial abundance in atmospheric environments is much lower than in other natural environments; therefore, accurate evaluation of viability of airborne bacteria remains difficult. In addition, new methods are required to simultaneously obtain bacterial viability and phylogenetic information.

We demonstrated that bacteria in Asian dust that had been transported long distances did not immediately influence the bacterial community in downwind areas. Furthermore, our findings suggest that bacterial communities may be affected more by ground environments along the transfer route and local environments than by the bacterial community in the dust itself. However, more severe occurrences of Asian dust in areas closer to the dust source may result in microbes in the dust having a greater impact on the indigenous bacterial community. The amount of Asian dust fallout is estimated to be 180 g m^−2^ year^−1^ in Beijing, China^26^ (500–2,500 km from the dust source region) and 0.005–0.05 g m^−2^ year^−1^ in Osaka, Japan^6^ (3,000–5,000 km from the dust source region). Accordingly, future studies of the bacterial community in downwind areas closer to the source are warranted to better assess the impacts of aeolian dust on public health and ecosystems.

## Materials and Methods

### Sample collection

Aerosol samples were collected from the rooftop of a building (ca. 20 m in height) at Osaka University in Osaka, Japan (latitude: N34°9′1.89″, longitude: E135°31′15.61″) using a high-volume air sampler (HV500R [SIBATA, Saitama, Japan]). The sampling point was located approximately 12 km from a major downtown area, and there were no industrial plants, agricultural fields or superhighways around the sampling point. Air samples were collected on 0.6 μm pore-size glass fiber filters at 500 L min^−1^. Aerosol particles from a total of 100 m^3^ of ambient air were collected during each sampling event (sampling time: 200 min). Furthermore, particle size distribution (>0.3 μm, >0.5 μm, >0.7 μm, >1.0 μm, >2.0 μm, and >5.0 μm) was determined using a particle counter (ARTI HHPC-6 [HACH, Tokyo, Japan]). Overall, 44 samples were obtained on different days between May 2013 and April 2015, including Asian dust days (20 May 2014, 27–31 May 2014, 1–3 June 2014, and 18 April 2015), all four seasons and after rainy days (Table 1). The occurrence of atmospheric Asian dust was confirmed using information from the Japan Meteorological Agency, LIDAR (Light Detection and Ranging) data from the Ministry of the Environment, Japan (http://www-gis5.nies.go.jp/eastasia/DustLider.php), and visibility at the sampling location (Supplementary Fig. S3).

The geographic origins of Asian dust collected in this study were determined by back trajectory analysis (http://ready.arl.noaa.gov/HYSPLIT.php), and we confirmed that the origin of all Asian dust samples was the Gobi Desert.

### DNA extraction

Aerosol samples collected on the glass filter were pulverized by bead-beating (EZ-Beads [EZ, Tokyo, Japan], 4,800 rpm, about 90 s). DNA was then extracted and purified using the method described by Tsai and Olson^27^. Extracted DNA was subsequently purified using a Wizard DNA Clean-Up System kit (Promega, Madison, WI, USA) and eluted with 50 μL of TE buffer (10 mM Tris-HCl and 1 mM EDTA [pH 8.0]).

### Estimation of bacterial abundance

To determine bacterial abundance, 16S rRNA gene was quantified by real-time PCR using a LightCycler (Roche Diagnostics, Mannheim, Germany). Real-time PCR was performed with eubacterial primer sets as described by Yamaguchi *et al.*^12^. A total of 1 × 10^1^ to 1 × 10^7^ copies per reaction of PCR products of *E. coli* W3110 were used as the DNA template to generate a standard curve for quantification of the 16S rRNA gene. The copy number of the 16S rRNA gene differed among bacterial phyla; therefore, bacterial abundance was calibrated based on the results of bacterial community composition analysis at the phylum level.

### Analysis of bacterial community composition

Two-step PCR was conducted to amplify the 16S rRNA gene for pyrosequencing^28^. Using this approach, tags and adapters were added in a second round of PCR amplification. The first PCR amplification was conducted using the universal primers 968F (AACGCGAAGAACCTTAC) and 1401R (CGGTGTGTACAAGACCC) to amplify a 434-bp fragment of the 16S rRNA gene between the V6 and V8 regions^29^. PCR amplification was performed using the reagents supplied with the AmpliTaq Gold kit (Applied Biosystems, Carlsbad, CA, USA). The reaction mixture contained 2.5 U mL^−1^ AmpliTaq Gold, 0.5 μ of each primer, 0.2 mM of each dNTP, 1.5 mM MgCl_2_, and 12.5 μg mL^−1^ 8-methoxypsoralen (Sigma Aldrich, St. Louis, MO, USA; dissolved in dimethyl sulfoxide) in 49 μL PCR buffer. A 1-μL DNA suspension was added after irradiation of the PCR mixture with ultraviolet light^30^. The reaction cycle consisted of an initial denaturing step at 94 °C for 9 min, followed by 10 cycles of denaturing at 95 °C for 1 min, annealing at 63–53 °C (decreased by 1 °C per cycle) for 1 min, and extension at 72 °C for 3 min. This was followed by 20 cycles of denaturing at 95 °C for 1 min, annealing at 53 °C for 1 min, and extension at 72 °C for 3 min, with a final extension step at 72 °C for 10 min. Primary PCR products were then purified using a MonoFas PCR Purification Kit (GL Sciences, Tokyo, Japan) and eluted with 40 μL of TE buffer. Next, a second round of PCR was performed as described above, except primers with an adapter and barcodes of 10 nucleotides in length were used. Furthermore, the number of PCR cycles was reduced to 20 (10 cycles of annealing at 63–53 °C and 10 cycles of annealing at 53 °C). Amplicon sequencing using Ion PGM (Thermo Fisher Scientific KK, Yokohama, Japan) was carried out at the Center for Medical Research and Education, Osaka University (Osaka, Japan).

Raw sequence data of the obtained amplicons were screened, trimmed, and filtered using the default settings of QIIME pipeline version 1.9.1 (http://qiime.org/), resulting in over 125,000 sequences across all samples (3,200 sequences per sample, on average). Total operational taxonomic units (OTUs), which were defined at the 97% nucleotide-sequence identity level using the UCLUST function of the QIIME software^31^, were identified in all sequences, with about 1,500 OTUs per sample on average being recovered. Beta diversity measures were also calculated. Differences in community composition of each sample were assessed graphically using the ordination method of non-metric MDS calculated based on the Euclidean distance.

Bacterial community composition was finally represented following calibration by copy number of the 16S rRNA gene of each phylum.

### Nucleotide sequence accession numbers

The sequences obtained from amplicon sequencing were deposited in the DNA Data Bank of Japan Sequence Read Archive under accession number DRA004472.

## Additional Information

**How to cite this article**: Park, J. *et al.* Investigation of bacterial effects of Asian dust events through comparison with seasonal variability in outdoor airborne bacterial community. *Sci. Rep.* **6**, 35706; doi: 10.1038/srep35706 (2016).

## Supplementary Material

Supplementary Information

## Figures and Tables

**Table 1:** Sample descriptions and associated physical characteristics of the atmosphere.

ID	Sampling date	Season	Start	End	Asian dust*	Weather	Temp. (°C)	Relative humidity (%)	Wind speed (m s^−1^)	Wind Direction
1	20130530	Spring	10:00	13:20	−		24.9	N. D.	3	SW
2	20130613	Summer	10:30	13:50	−		18.4	N. D.	3	SW
3	20130627	Summer	9:10	12:30	−	After rainy	26.9	N. D.	3	NW
4	20130722	Summer	10:00	13:20	−		31. 5	N. D.	4	SW
5	20130805	Summer	10:00	13:20	−		33.4	N. D.	3	SW
6	20130820	Summer	10:00	13:20	−		34.7	N. D.	3	SSW
7	20130827	Summer	10:00	13:20	−		30.2	46	3	NW
8	20130903	Fall	10:00	13:20	−		27.5	72	3	ENE
9	20130911	Fall	10:00	13:20	−		30.1	52	2	SSW
10	20130914	Fall	10:20	13:40	−		30.9	59	6	E
11	20130918	Fall	10:00	13:20	−		27.5	50	2	NE
12	20131002	Fall	10:00	13:20	−		25.4	56	2	NW
13	20131017	Fall	10:00	13:20	−	After rainy	19.2	39	6	N
14	20131105	Fall	10:40	14:00	−		18.1	36	3	N
15	20131118	Fall	10:10	13:30	−	After rainy	12.7	31	5	WSW
16	20131203	Winter	10:00	13:20	−		11.9	42	3	SW
17	20140129	Winter	15:30	19:00	−		9.7	24	1	SE
18	20140331	Spring	9:40	13:00	−	After rainy	15.6	23	5	N
19	20140423	Spring	8:40	12:00	−		19.4	31	3	SW
20	20140516	Spring	11:50	15:10	−	After rainy	22.6	36	4	SSW
21	20140517	Spring	12:00	15:20	−		22.2	26	3	NW
22	20140608	Summer	10:40	14:00	−	After rainy	27.3	54	3	SSW
23	20141111	Fall	10:00	13:20	−		17.4	N. D.	1	ESE
24	20141127	Fall	11:00	14:20	−	After rainy	17.0	N. D.	5	N
25	20141217	Winter	10:00	13:20	−	After rainy	3.5	29	6	W
26	20141224	Winter	10:00	13:20	−		8.7	47	2	SW
27	20150114	Winter	10:00	13:20	−		6.5	44	2	ENE
28	20150128	Winter	10:10	13:30	−		5.6	29	6	NNW
29	20150212	Winter	10:00	13:20	−		7.0	47	3	WSW
30	20150227	Winter	10:00	13:20	−	After rainy	8.2	29	5	N
31	20150311	Spring	10:00	13:20	−	After rainy	5.8	30	5	WSW
32	20150416	Spring	11:00	14:20	−	After rainy	18.9	42	4	SW
33	20150417	Spring	10:10	13:30	−		17.2	31	2	N
34	20150423	Spring	11:10	14:30	−		20.7	27	3	S
35	20140520	Spring	10:00	13:20	+		27.2	35	2	N
36	20140527	Spring	11:40	15:10	+		24.1	44	2	S
37	20140528	Spring	10:20	13:40	+		22.4	32	2	SW
38	20140529	Spring	13:10	16:30	+		25.9	30	2	S
39	20140530	Spring	11:40	15:00	+		26.6	35	3	SW
40	20140531	Spring	12:00	15:20	+		28.1	45	3	S
41	20140601	Summer	10:30	13:50	+		28.3	44	3	SW
42	20140602	Summer	10:30	13:50	+		30.0	39	4	SW
43	20140603	Summer	10:30	13:50	+		29.4	41	4	SW
44	20150418	Spring	10:45	15:05	+		19.3	24	3	WSW

^*^Determined by information obtained from the Japan Meteorological Agency, LIDAR, and visibility at the sampling site.
