# Supplementary material for: Investigation of bacterial effects of Asian dust events through comparison with seasonal variability in outdoor airborne bacterial community
Source: Sci Rep. 2016 Oct 20;6:35706. doi: 10.1038/srep35706 (PMC5071759; doi:10.1038/srep35706)
Supplement: Supplementary Information [file srep35706-s1.pdf]

Supplementary Information

Investigation of bacterial effects of Asian dust events through comparison with seasonal variability in outdoor airborne bacterial community

Jonguk Park[1], Tomoaki Ichijo[1], Masao Nasu[1,2] and Nobuyasu Yamaguchi*[1,3]

[1] Graduate School of Pharmaceutical Sciences, Osaka University, 1-6 Yamada-oka, Suita, Osaka 565-0871, Japan

[2] Faculty of Pharmacy, Osaka Ohtani University, 3-11-1 Nishikiori-kita, Tondabayashi, Osaka 584-8540, Japan

[3] Osaka Prefectural Institute of Public Health, 1-3-69 Nakamichi, Higashinari, Osaka 537-0025, Japan

*Corresponding should be addressed to N. Y.

(nyyamaguchi@iph.pref.osaka.jp)

Supplementary Figures 1-3

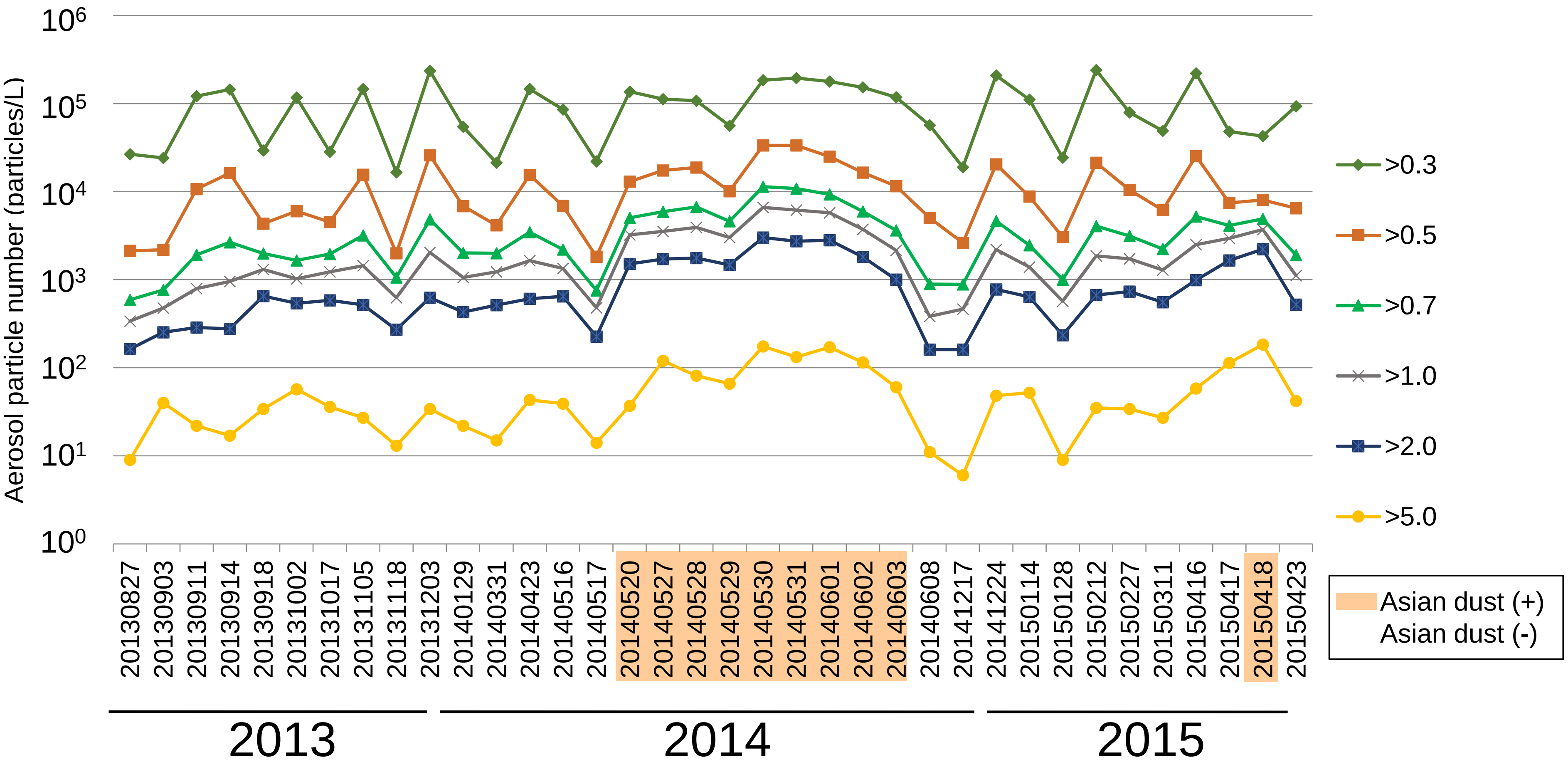

Supplementary Fig. S1. Particle size distribution of aerosols in outdoor environments.

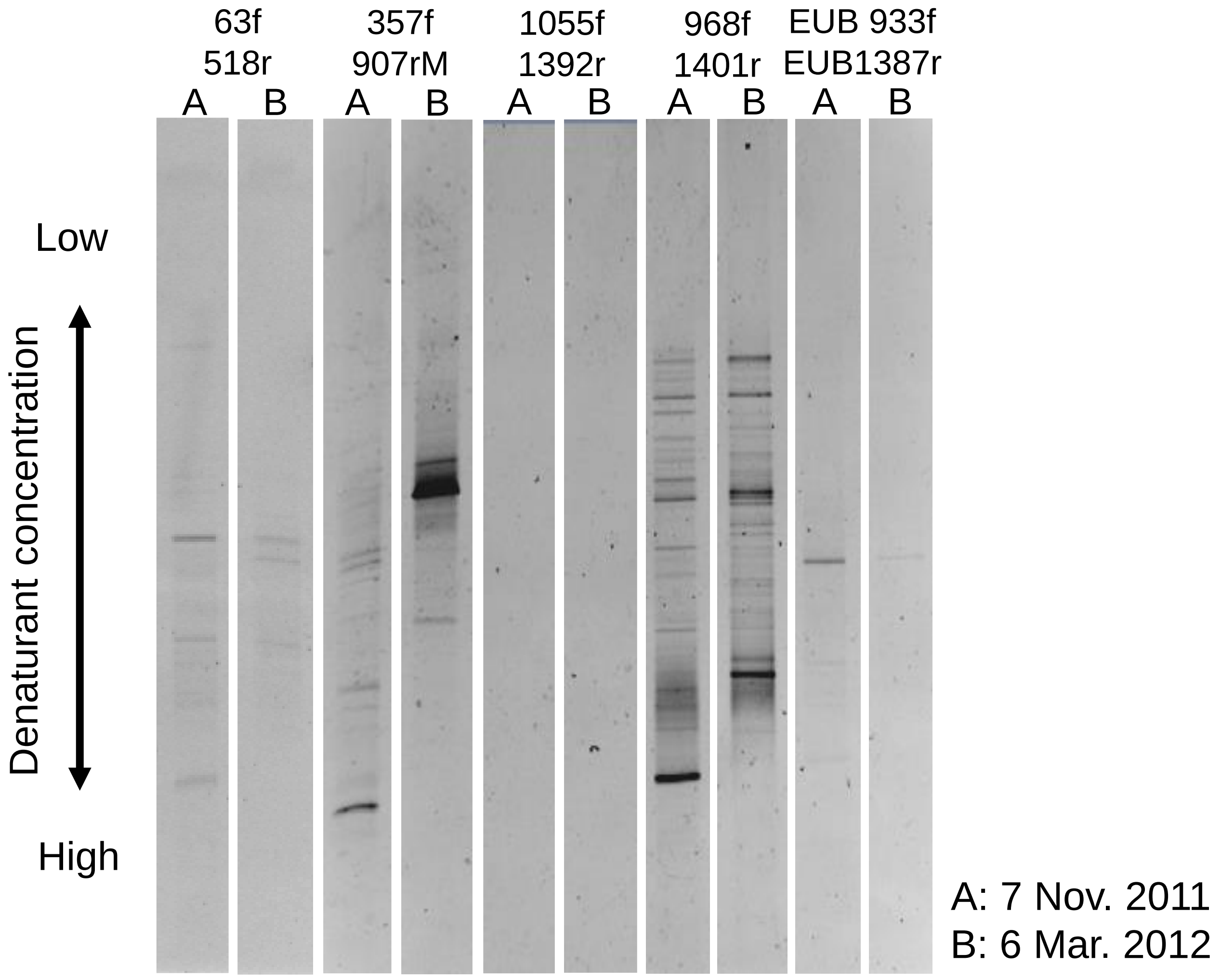

Supplementary Fig. S2. Selection of proper primers for amplicon sequencing using PCR-DGGE. Aerosol samples were collected outdoors using a high-volume air sampler.

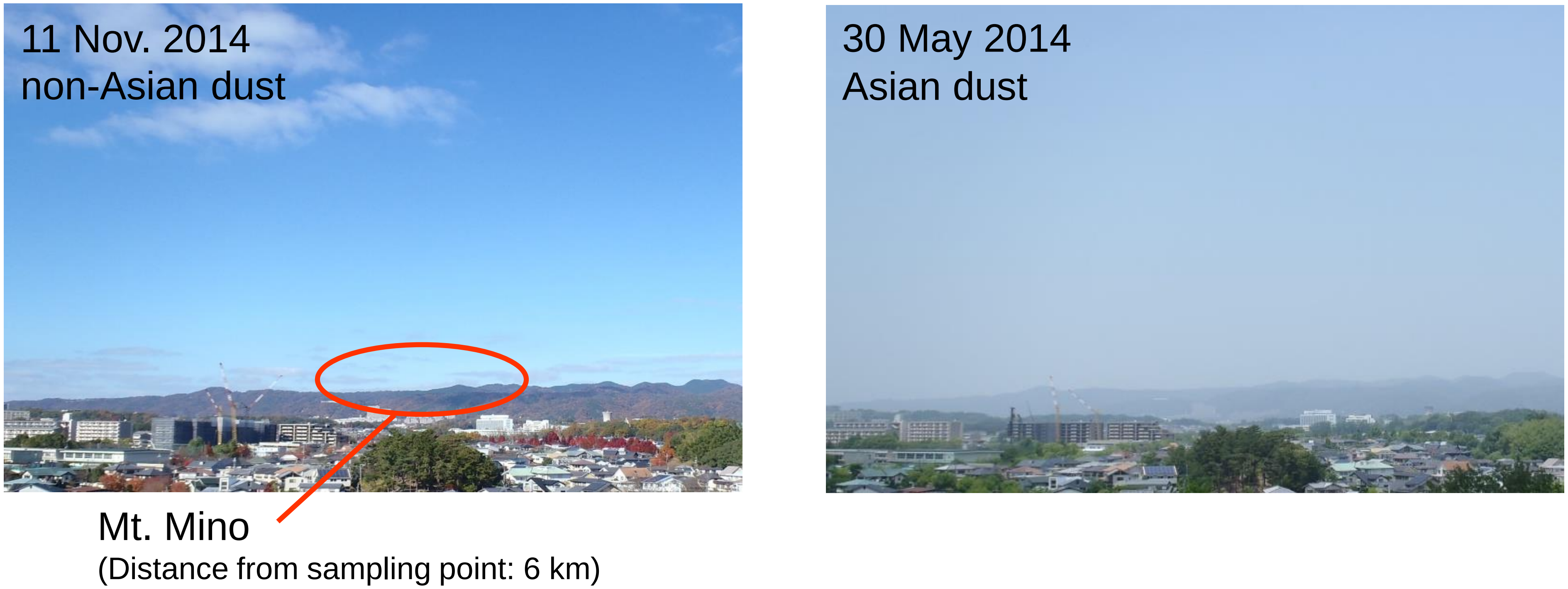

Supplementary Fig. S3. Confirmation of Asian dust events based on visibility from the sampling point (ca. 20 m in height).
